# Factors Determining the Quality of Life of Polish Women during Menopause Based on the Menopause-Specific Quality of Life Questionnaire

**DOI:** 10.3390/healthcare11081173

**Published:** 2023-04-19

**Authors:** Agnieszka Bień, Magdalena Korżyńska-Piętas, Marta Zarajczyk, Mariusz Wysokiński, Iwona Niewiadomska, Krzysztof Jurek, Ewa Rzońca

**Affiliations:** 1 Chair of Obstetrics Development, Faculty of Health Sciences, Medical University of Lublin, 20-081 Lublin, Poland; 2 Chair of Nursing Development, Faculty of Health Sciences, Medical University of Lublin, 20-081 Lublin, Poland; 3 Department of Social Psychoprevention, John Paul II Catholic University of Lublin, 20-950 Lublin, Poland; 4 Institute of Sociological Sciences, John Paul II Catholic University of Lublin, 20-950 Lublin, Poland; 5 Department of Obstetrics and Gynecology Didactics, Faculty of Health Sciences, Medical University of Warsaw, 00-575 Warsaw, Poland

**Keywords:** menopause, quality of life, menopause-specific quality of life questionnaire, validity, Poland

## Abstract

Background: The aim of the study was to present the process of cultural adaptation to Polish conditions and the validation of a scale assessing the quality of life of Polish women during the menopause and to identify the factors determining this quality of life. Methods: The research tools were the menopause-specific quality of life (MENQOL) questionnaire and a standardized interview questionnaire comprising questions on the participants’ characteristics. The study involved 516 women using health care services who had symptoms caused by the menopause. Results: The value of the Cronbach’s alpha coefficient was 0.923. The discriminative power coefficients of all the questionnaire items were higher than 0.3. The study confirmed the validity and internal consistency of the Polish version of the MENQOL questionnaire for measuring the quality of life of postmenopausal women, suggesting that the tool can be used for screening menopausal symptoms in women. There was a relationship between general quality of life and age (*p* = 0.002), marital status (*p* < 0.001), education (*p* = 0.021), the impact of professional work (*p* < 0.001), the impact of physical activity (*p* < 0.001) and the impact of social life (*p* < 0.001). Conclusion: In the group of women who took part in the study, the authors observed a lower quality of life during menopause reported by older women who were married/in a stable relationship, with no formal education (no formal education) and who, according to their subjective assessment, negatively evaluated the impact of the accompanying menopause-related symptoms on their work, physical activity and social life.

## 1. Introduction

The perimenopausal period in a woman’s life is a time that involves changes in the female body regarding premenopause, menopause and postmenopause. The premenopausal period is characterized by a decrease in the secretion of steroid hormones, the disappearance of fertility and the appearance of various symptoms, along with irregular bleeding [1]. These changes lead to the menopause, which, according to the World Health Organization (WHO), marks the physiological, permanent cessation of menstruation as a result of the cessation of the ovarian function [2]. It is estimated that menopause occurs on average between the ages 45 and 55 [1]. The peri-menopausal period is associated with changes in estrogen and progesterone concentrations. The resulting hormonal changes affect women’s psychological and somatic functioning and can, therefore, also make it difficult to fulfil social, professional and family roles. Hot flushes, excessive sweating, sleep disturbance, irritability, an increased urge to urinate, sexual dysfunction and others are the most common complaints that occur during this period and hinder daily functioning. Although it has been observed that women’s own perceptions and the perceptions of peri-menopausal symptoms vary widely, such changes have been shown to have an adverse impact on women’s quality of life [3,4,5]. The peri-menopausal period is also often marked by the onset or exacerbation of diseases, such as diabetes, cardiovascular disease, osteoporosis and atherosclerosis, which undoubtedly affect women’s quality of life [6,7].

Quality of life (QoL) is an interdisciplinary concept used in various sciences and disciplines, including medicine, psychology, nursing, sociology, politics or economics. Quality of life in medicine serves to indicate the patient’s needs and to help make therapeutic decisions, taking into account the complaints experienced and their impact on the patient’s life. Due to the multifaceted nature of the concept of quality of life, particular attention should be paid to the precise selection of the research tools for assessing quality of life in individual studies [8]. Since the middle of the 20th century, the concept of the quality of life has been of interest to many researchers and clinicians dealing with various health and physiological problems. The peri-menopausal period has been no exception to this due to the increase in life expectancy, the importance for women’s health and the perception that this period of life is as important as the reproductive period. It is estimated that most women live one-third of their lives in the peri-menopausal period, which is why, among other things, it is very important to pay attention to women’s health during this period of their life in order to be able to provide them with the highest possible quality of life [5,9].

### Purpose of the Study

The aim of the study was to present the process of cultural adaptation to Polish conditions and the validation of a scale assessing the quality of life of Polish women during the menopause and to identify the factors determining this quality of life.

## 2. Materials and Methods

### 2.1. Study Design and Participants

A questionnaire technique and a diagnostic survey method was used in the study. The research tools were the menopause-specific quality of life (MENQOL) questionnaire and a standardized interview questionnaire comprising questions on the participants’ characteristics. Permission to use the Polish-language version of the questionnaire was obtained from ePROVIDE^TM^, Mapi Research Trust. The menopause-specific quality of life (MENQOL) questionnaire by Hilditch et al. consists of 29 sub-items with a 7-point scale from 0 to 6. Each of the sub-items contains a symptom that may occur during the menopause and includes four domains: vasomotor (items 1–3), psychosocial (items 4–10), physical (items 11–26), and sexual (items 27–29). Respondents indicate whether a particular problem has affected them in the past month. If so, they indicate to what extent the problem has bothered them on a scale from 0 to 6, where 0 means not bothered at all and 6 means bothered a lot. With increasing MENQOL scores, the levels of bother experienced due to the symptom are increased as well. The reliability of the questionnaire as measured by the α-Cronbach internal consistency coefficient was 0.8 [10].

### 2.2. Subjects

**Figure 1:**
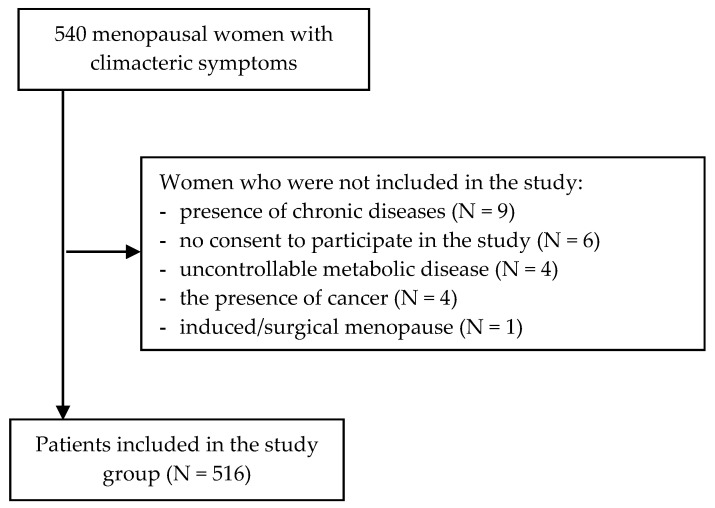
Flow diagram of exclusions and final analytic sample.

The study was conducted between April 2020 and March 2021, and involved 516 women using health care services (primary care, specialist outpatient care and inpatient/hospital care) in the Lubelskie Voivodeship (Poland). The inclusion criteria were: the presence of menopausal symptoms, aged between 41 and 60 years, no use of available treatments for menopause-related symptoms (hormone therapy, selective estrogen receptor modulators, aromatase inhibitors and soybean extracts) by the respondents in the last three months before the study, and the native language was Polish. The exclusion criteria for the study were: induced/surgical menopause (hysterectomy, ovarian excision, radiotherapy and chemotherapy), and the presence of chronic diseases, such as kidney impairment, immunodeficiency, or cardiovascular disease, mental illness, untreated thyroid disease, a cancer diagnosis, those with a neurological disease, and uncontrollable metabolic disease (Figure 1). Of the 540 survey questionnaires distributed, 516 correctly completed questionnaires were further analyzed and the success rate of the data obtained was 95.55%. The study group was a research cohort.

Menopausal status was defined in accordance with the WHO’s classification. To elucidate this distribution, women with regular menstrual bleeding during the last year were classified as premenopausal, those with irregular bleeding during the last 12 months as perimenopausal and those with amenorrhea during the last year as menopausal. Finally, women were classified as postmenopausal, if they had no menstrual bleeding from 1 year and above [2].

The study was approved by the Bioethics Committee of the Medical University of Lublin (approval no. KE-0254/257/2020). The respondents were informed that participation was voluntary, and that the study results were anonymous and would be used exclusively for research purposes.

### 2.3. Statistical Analysis

The Cronbach’s alpha coefficient was used to assess reliability by testing the internal consistency of the scale. The sampling adequacy was examined using the Kaiser–Mayer–Olkin test. The theoretical relevance was assessed using exploratory factor analysis, which was conducted using the principal components’ method with Oblimin simple rotation with Kaiser normalization. The reliability of the tool was estimated on the basis of the discriminant power values of the items forming the highlighted dimensions. Pearson’s r correlation coefficient was used to assess the correlation of the subscales with the total score. The influence of selected sociodemographic factors on women’s attitudes towards breastfeeding was assessed using Student’s *t* test and one-way analysis of variance (ANOVA). A significance level of *p* < 0.05 was adopted. Statistical analyses were performed using IBM SPSS Statistics (PS IMAGO) v. 26 computer software.

## 3. Results

The mean age of the women was 50.24 years (SD = 3.96), the majority of respondents were married/in a stable relationship (81.4%), had a university degree (64.0%), were working (81.4%), rated their family wealth as average (88.2%), had a BMI indicating that they were overweight (39.7%), subjectively judged that the symptoms associated with the menopause have a negative impact on their work (41.1%), on their physical activity (46.9%) but did not affect their social life (59.5%), as shown in Table 1.

### 3.1. Factor Structure of the Polish Version of the MENQOL Scale

**Figure A1:**
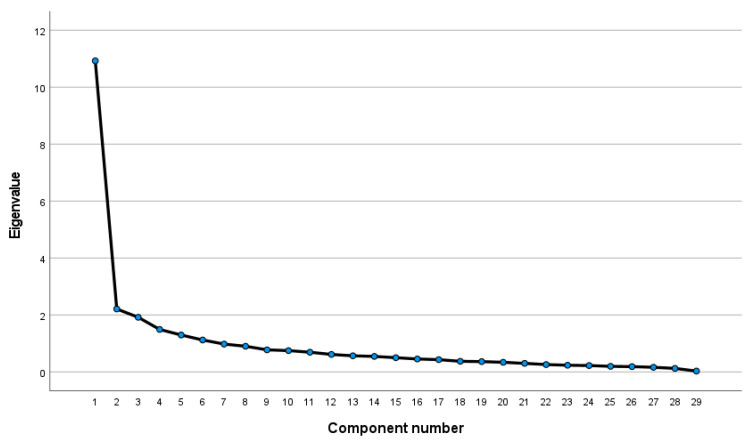
Scree plot of the 29-item MENQOL.

Principal components analysis (PCA) with oblique promax rotation was performed for the baseline MENQOL administration, to determine whether the latent item structure mirrored the four domains specified in the tool’s construction. Promax rotation was utilized due to the high number of component inter-correlations, indicating that the factors would likely be correlated. A Kaiser–Meyer–Olkin (KMO) statistic of 0.874 indicated that factor analysis was appropriate for the data and Bartlett’s test of sphericity was significant, suggesting the absence of multicollinearity (Ch square = 9869.907; df = 406; *p* < 0.001). The scree plot suggested the extraction of four factors (Appendix A, Figure A1).

Six eigenvalues exceeded one and explained 65.42% of the variance. Item loadings in the component, pattern and structure matrix with six extracted components revealed the emergence of t four strong components that explained under 50% of the variance. Two eigenvalues explained less than 5% of the variance. The items were considered representative of a component if their individual item loading was ≥0.50.

Regarding the vasomotor domain, items 1–3, correspond to the original vasomotor subscale. All the items loaded above 0.70 on a single component with no identified cross-loadings (items that loaded ≥0.30 on two or more components). Regarding the psychosocial domain aspects, items 4–10, correspond to the original psychosocial subscale. In the Polish version, four items were included in this subscale: “Feeling tired or worn out”; “Difficulty sleeping”; “Decrease in stamina (energy to keep going)”; “Lack of energy”. All loaded above 0.5 on a single component with no cross-loadings. Regarding the physical domain, in the Polish version, six items were included in the physical subscale. The original version contained 16 items. All loaded above 0.5 on a single component with no cross-loadings. Regarding the sexual domain, items 27–29, correspond to the original sexual subscale. All the items loaded above 0.70 on a single component with no identified cross-loadings. Items 11, 12, 15, 16, 23 and 24 are not shown here due to loadings on non-extracted components (i.e., components which did not explain a significant amount of variance or did not have ≥0.5 item loadings), as shown in Table 2.

### 3.2. Reliability

To assess the extent to which each subscale of the MENQOL measured a similar construct, internal consistency reliability with Cronbach’s α was computed for each subscale at the baseline. The Cronbach’s alpha coefficient for the Polish MENQOL for each domain was, respectively: vasomotor 0.790, psychosocial 0.924, physical 0.820 and sexual 0.813 (Table 3).

In Table 4, the basic characteristics for each item with the discriminatory power index of the item and the calculated reliability index (using the Cronbach’s alpha method) for the whole scale are shown. The Cronbach’s alpha of the whole scale (Polish version) was meaningful (0.923). The discriminative power coefficients of all the questionnaire items were higher than 0.3.

The statistical analysis performed showed significant positive correlations between the individual MENQOL subscales and the total score. The correlations had values ranging from 0.367 to 0.789 (Table 5).

The analysis showed a negative correlation between age and the psychosocial domain (*p* < 0.001), physical domain (*p* = 0.002), sexual domain (*p* = 0.031) and women’s total QoL (*p* = 0.002). Married women and women staying in a stable relationship had higher scores in the physical (*p* = 0.040), sexual (*p* < 0.001) and total QoL domains than women who were single. Those with tertiary education had lower symptom severity in the vasomotor (*p* < 0.001) subscale than women with primary or secondary education. In the case of the physical (*p* = 0.006) and global QoL scores (*p* = 0.021) subscales, women with tertiary education had lower symptom severity than women with primary education. Respondents who did not work had lower physical QoL than those who worked (*p* = 0.012). There was a positive correlation between material status and the vasomotor domain (*p* = 0.006). The women with normal weight had lower symptom severity in the vasomotor (*p* = 0.003) and physical (*p* < 0.001) domains than women who were overweight or obesity. In addition, women who were underweight had higher symptom severity in the psychosocial subscale (*p* = 0.041) than women who were overweight. Higher scores in all MENQOL domains, as well as in global quality of life, were obtained by respondents according to whom menopause-related symptoms affected their work (*p* < 0.001), their physical activity (*p* < 0.001), as well as their social life (*p* < 0.001), as shown in Table 6.

## 4. Discussion

Quality of life during the menopause can be discussed according to various aspects, starting from the subjective perception of one’s position in life, to health status specific to the period of life, to looking through the lens of specific physiological changes and their associated consequences. The first aspect analyzed was the evaluation of the psychometric properties of the Polish version of the menopause-specific quality of life (MENQOL) questionnaire by Hilditch et al. used in our study [10]. Principal components analysis (PCA) showed a four-factor model. Inter-scale correlation analysis showed that all 29 scale items were positively correlated with each other. The correlations ranged from 0.381 to 0.764, indicating that the items on the measure provide an accurate assessment of the quality of life of menopausal women. The MENQOL questionnaire has been validated in many countries, it has shown good predictive validity and excellent internal consistency of the Cronbach’s alpha for the entire MENQOL scale [11,12,13,14,15,16]. The reliability of the Polish version of the entire MENQOL scale, as measured by the Cronbach’s alpha, was 0.923, comparable to the Persian (0.9), Malaysian (0.9), Emirati (0.941), Serbian (0.957) or Korean (0.97) versions of the questionnaire [12,13,14,15,16]. The study obtained satisfactory and high levels of internal consistency for the individual scales, indicating their reliability. The reliability coefficients for the Polish adaptation in each domain were: vasomotor 0.790, psychosocial 0.924, physical 0.820 and Sexual 0.813. In comparison, in the study by Gazibar et al. [15] they were: vasomotor 0.917, psychosocial 0.907, physical 0.928 and sexual 0.913. In the study by Yerra et al. [17], conducted among Indian women, the reliability coefficients were: vasomotor domain 0.880, psychomotor domain 0.661, physical domain 0.821 and sexual domain 0.833. 

Using factor analysis, the authors found that the two domains, vasomotor and sexual, were the same as the original structure of the MENQOL scale. The psychosocial factor was similar to the original questionnaire. The Polish version of the psychosocial subscale included an additional four items that were originally in the physical domain: “Feeling tired or worn out”; “Difficulty sleeping”; “Decrease in stamina (energy to keep going)”; “Lack of energy”. These items in the Polish version are well-matched (all loaded above 0.5 on a single component with no cross-loadings). The rationale for this may be that fatigue, lack of energy and lack of sleep in a person’s life have an impact on more than just the physical aspect, but also in the psychological sphere. Individuals who are more tired are also significantly less likely to be willing to enter into new and maintain existing social relationships [18]. The results of the analysis showed factor loadings for six items from the physical domain: “Flatulence (wind) or gas pains”, “Aching in muscle and joints”, “Aches in back of neck or head”, “Decrease in physical strength”, “Feeling bloated” and “Low backache”. These low findings did not explain a significant amount of variance or did not have ≥0.5 item loadings. These symptoms can be classified as physical symptoms less characteristic of the menopausal period. Justification can also be found in the WHO’s definition of quality of life, which states that it is an individual’s perception of his or her position in life in the context of the cultural and value systems in which they live and in relation to their goals, expectations, standards and concerns [19].

The second aim of our study was to assess the quality of life of Polish women during the menopause and to identify the determinants of it. Nowadays, the average life expectancy of women is about 80 years and is steadily increasing, with one-third of their lives falling into the postmenopausal period, which occurs around the age of 50 [8,20,21]. Moreover, women in their 50s are required to be fully physically, intellectually and socially active. The results of our study, as well as reports from other countries, confirm that as women’s age increases, the severity of menopausal symptoms reduces their overall quality of life [6,22,23]. We have found that the older the respondents, the lower their assessed quality of life in the psychosocial, physical and sexual domains. Willians et al. [24], Som et al. [25] and Shoberi et al. [26] observed that aging women had lower QOL scores, except for respondents in the 60 to 65 years of age range, whose scores again increased in the psychosocial, physical and sexual domains. Probably, postmenopausal women have a greater ability to cope with or are less affected by the discomforts which occurred during the menopause.

The need for family support and a sense of emotional bonds also increases with age [23]. The severity of menopausal complaints may be related to the partner’s attitude towards the changes that menopause entails [27]. Analysis of our research has shown that women in a relationship rate their mental and physical health and, thus, their quality of life, worse than those who are single. Perhaps those who are in a relationship feel more embarrassed in front of their partner about menopause-induced symptoms. In contrast, studies by other authors suggest that married women have a more positive attitude towards the menopause than divorced/single women and enjoy a better quality of life [28,29].

The educational level is one of the important determinants of quality of life in menopausal women [6,30,31,32]. The results of our study showed that respondents with a university degree rated their overall quality of life better, as well as their quality of life in the vasomotor and physical domains. These results are consistent with those of other researchers representing different regions of the world and different cultures. Higher education also translated into higher quality of life for postmenopausal women living in Iran [6,30]. Educational level was the only common significant factor affecting the QoL of Mexican and Spanish climacteric women respondents, according to a study by Larroy et al. Unlike marital status and socioeconomic level, which only affected the QoL of Mexican women respondents, occupational activity was not significant for the QoL of both groups [31]. The association between educational level and QoL could be explained by the influence of higher intellectual potential in understanding the changes that occur during the menopause and during the ageing process, as well as higher health awareness and the adoption of pro-health behaviors by women with a university degree.

The results of our study showed that respondents who were economically active rated their quality of life in the physical sphere higher than those who were unemployed. On the other hand, respondents who claimed that menopause-related symptoms had a negative impact on their professional work rated their global quality of life worse, as well as their quality of life in individual domains, compared to respondents who did not feel such a negative impact of these symptoms on their professional work. Barati et al. [6] emphasize that active women may have better access to health care and, thus, better manage menopausal symptoms. 

The results of our own study showed that the higher the socioeconomic status, the higher the quality of life was for the women studied in the vasomotor domain. Relevant in this regard is the study by Mirhaghjou et al. [30], which showed that having a university degree and being employed were associated with better quality of life. Having a university degree combined with being economically active can be seen as being associated with having a higher income, at the same time as having greater access to health care services or greater awareness of potential ways to manage menopausal symptoms.

In our study, obese and overweight women showed the lowest quality of life in the vasomotor domain. For the psychosocial domain, underweight and obese women presented the lowest quality of life, while in the physical domain, underweight and overweight women presented the lowest quality of life. Similar conclusions were presented by Mirhaghjou et al. [30] and Obara-Gołębiowska [33], showing that women with normal BMI reported a higher quality of life compared to overweight and obese women. In contrast, Ghazanfarpour et al. [12] found that obesity did not affect perceptions on the overall quality of life, while showing that women with obesity showed poorer physical functioning.

Previous studies on the impact of physical activity on the functioning of women during menopause, demonstrates a beneficial effect on reducing perimenopausal complaints and improving the quality of life [34,35]. The results of our study show that women who declared the impact of menopausal symptoms on their physical activity, had a lower overall quality of life and a lower quality of life in every sphere. Other researchers have had the same opinion, showing that increased physical activity translates into the alleviation of unpleasant menopausal symptoms, thus resulting in an improvement in women’s quality of life [34,35,36,37]. 

Satisfactory interpersonal relationships may help to reduce the negative impact of the symptoms accompanying the menopause [18]. In our study, women who declared that perimenopausal complaints affected their social life showed lower overall quality of life and lower quality of life in each of the MENQOL domains, compared to women who did not perceive such an impact. Positive interpersonal relationships and support help to reduce negative thoughts, feelings of guilt and minimize the risk of depression, thereby positively influence women’s quality of life [18,36].

Differences between the scores in individual QoL domains and in different regions of the world may be due to existing cultural diversity. In European countries, women find menopausal symptoms, especially vasomotor symptoms, more problematic, which may be influenced by their education and work activity. This demonstrates the role of education and work status in the experience of menopausal symptoms. In Arab countries, menopause is considered the ‘age of despair’, meaning the ‘end of life’ for women, as they no longer have the capacity to reproduce. In contrast, Indian women perceive menopause as a natural period in a woman’s life, accompanied by physical symptoms resulting from the ageing process, while symptoms related to the sexual sphere are hidden, which may be because they are treated as a taboo and are associated with cultural beliefs [17,38]. 

A tool with very good psychometric properties was obtained as a result of work on the Polish version of the questionnaire assessing the quality of life of women during the menopause. This will allow for the possibility of comparing the obtained results on an intercultural level and for comparing the experiences of menopausal women around the world. Due to the concise structure of the questionnaire, it may be particularly useful in time-consuming studies involving multiple tools. 

In conclusion, it appears that menopausal symptoms are caused by a combination of physical changes, the influences of sociodemographic, sociocultural factors and individual perception. The ageing of the population in Poland is increasing the number of women experiencing the menopause and, therefore, this period requires a more in-depth look by the health care system. The population of women at this age should become the main target of preventive and health policy programs aimed at women during this period. The economic aspect should also be taken into account. Additional costs arise from the need to alleviate symptoms (hormone replacement therapy, psychotherapy, acupressure, herbal compounds, acupuncture, massage, yoga and lifestyle changes), as well as from the long-term treatment for the consequences of sex hormone deficiency, from follow-up visits, laboratory tests or reduced productivity at work [39]. The MENQOL questionnaire allows the assessment of the quality of life of menopausal women, and the results allow for providing support and care to women with a lower quality of life demonstrated in individual domains.

### Strengths and Weaknesses of the Study

A strength of our study is the criteria used to select the study group of women, from which we excluded those with medical conditions whose symptoms may exacerbate menopausal symptoms or may be identified with the menopause. We assessed quality of life using a standardized tool, which is also a strength of our study. On the other hand, a limitation to our study is that it has a cross-sectional design, which does not permit assessment of temporal or causal relationships between the variables. During the interview, we did not ask respondents about their history of hormonal contraceptive use, the time and type of work they held or the support they received from loved ones, all of which may affect the quality of life of menopausal women. We are also aware that subjective variation in symptom severity may limit the study results. The question format of the MENQOL questionnaire assesses only those aspects of the menopausal experience that are perceived negatively by women, but these negative aspects are reported to be more likely to respond to change than positively perceived items in terms of their impact on the quality of life.

## 5. Conclusions

With age, respondents’ quality of life assessment declines especially in the psychosocial, physical and sexual domains.

Poorer material status, no formal education, higher body mass index and a negative assessment of the impact of accompanying menopause-related symptoms on their work, physical activity and social life have a statistically significant association with higher scores in the vasomotor domain.

Advanced age, being in a relationship/marriage, declaring less than a university degree, not being economically active, being underweight and negatively assessing the impact of menopausal symptoms on professional work, physical activity and social life are all factors that reduce respondents’ quality of life in the physical domain.

Lower quality of life in the sexual domain is reported by women living in rural areas, married or staying in a stable partnership, expressing the opinion that menopause-related symptoms affect their work, physical activity and social life.

The Polish version of MENQOL is a reliable and appropriate tool for measuring the quality of life of menopausal women and shows very good psychometric properties and construct validity.

## Figures and Tables

**Table 1:** Sociodemographic characteristics of the respondents.

Characteristics of the Group		*N* (%)
Mean age/years (M, SD)		50.24 (3.96)
Relationship status	Single	96 (18.6)
	Married/in a stable relationship	420 (81.4)
Education	Primary	40 (7.8)
	Medium	146 (28.3)
	Higher	330 (64.0)
Professional status	Working	420 (81.4)
	Not working	96 (18.6)
Your family’s wealth	Very rich/rather rich	50 (10.1)
	Average	455 (88.2)
	Rather poor/poor	9 (1.7)
BMI (kg/m^2^)	Underweight	6 (1.2)
	Correct	195 (37.8)
	Overweight	205 (39.7)
	Obesity	110 (21.3)
Impact of symptoms on professional work	Yes	212 (41.1)
	No	186 (36.0)
	Not applicable	118 (22.9)
Impact of symptoms on physical activity	Yes	242 (46.9)
	No	217 (42.1)
	Not applicable	57 (11.0)
Impact of symptoms on social life	Yes	150 (29.1)
	No	307 (59.5)
	Not applicable	59 (11.4)

M—mean; SD—standard deviation; BMI—body mass index.

**Table 2:** Factor structure of the Polish version of the MENQOL scale.

MENQOL Domains		Items	Extracted Component			
			Component 1 (Psychosocial)	Component 2 (Physical)	Component 3 (Vasomotor)	Component 4 (Sexual)
Vasomotor	1	Hot flushes or flashes	−0.065	0.070	0.745	0.004
	2	Night sweats	−0.081	−0.109	0.802	0.173
	3	Sweating	−0.021	−0.016	0.702	0.122
Psychosocial	4	Dissatisfaction with my personal life	0.686	−0.204	0.045	0.294
	5	Feeling anxious or nervous	0.809	−0.168	0.128	0.075
	6	Poor memory	0.645	0.138	−0.172	0.142
	7	Accomplishing less than I used to	0.818	0.162	−0.266	−0.055
	8	Feeling depressed, down or blue	0.925	−0.216	0.019	0.008
	9	Being impatient with other people	0.688	−0.162	0.049	0.267
	10	Feelings of wanting to be alone	0.625	−0.126	0.087	0.184
Physical	11	Passing gas or gas pains	0.178	0.238	0.422	−0.077
	12	Aching in muscles and joints	0.364	0.263	0.252	−0.168
	13	Feeling tired or worn out	0.762	0.061	0.154	−0.051
	14	Difficulty sleeping	0.786	0.185	−0.015	−0.219
	15	Aches in back of neck or head	0.459	0.048	0.258	−0.170
	16	Decrease in physical strength	0.306	0.076	0.252	0.048
	17	Decrease in stamina (energy to keep going)	0.874	0.100	−0.039	−0.109
	18	Lack of energy	0.835	0.112	−0.071	−0.029
	19	Dry skin	0.159	0.581	−0.217	0.314
	20	Weight gain	−0.113	0.776	0.023	−0.031
	21	Increased facial hair	0.009	0.907	−0.078	0.127
	22	Changes in appearance, texture or tone of my skin	0.005	0.764	0.018	−0.004
	23	Feeling bloated	0.058	0.085	0.304	−0.073
	24	Low backache	0.105	0.105	0.433	−0.046
	25	Frequent urination	−0.089	0.611	0.086	0.186
	26	Involuntary urination when laughing or coughing	0.014	0.533	0.220	0.018
Sexual	27	Decrease in my sexual desire	0.015	0.174	0.034	0.780
	28	Vaginal dryness	−0.147	0.154	0.169	0.703
	29	Avoiding intimacy	0.141	−0.016	−0.007	0.768

**Table 3:** Internal consistency reliability.

Subscale Domains	Cronbach’s Alpha *	Item Number(s) Added/Deleted	Cronbach’s Alpha **
Vasomotor	0.790	-	0.790
Psychosocial	0.895	13; 14; 17; 18	0.924
Physical	0.896	11;12; 15; 16; 24; 26	0.820
Sexual	0.813	-	0.813

* Original set of items; ** Polish version.

**Table 4:** Assessment of the reliability of the Polish version of the MENQOL scale.

Items	Scale Average after Removal of Items	Scale Variance after Removal of Items	Item Total Correlations	Cronbach’s α if Item Deleted
Hot flushes or flashes	95.39	1022.845	0.462	0.921
Night sweats	95.62	1018.776	0.476	0.921
Sweating	95.39	1019.264	0.485	0.921
Dissatisfaction with my personal life	95.61	998.157	0.646	0.918
Feeling anxious or nervous	94.34	996.194	0.708	0.917
Poor memory	94.57	1011.438	0.599	0.919
Accomplishing less than I used to	94.33	1020.447	0.593	0.919
Feeling depressed, down or blue	94.88	993.254	0.672	0.917
Being impatient with other people	94.47	1001.729	0.671	0.918
Feelings of wanting to be alone	94.61	1001.113	0.601	0.919
Feeling tired or worn out	93.67	1003.614	0.764	0.917
Difficulty sleeping	94.68	1015.291	0.488	0.921
Decrease in stamina (energy to keep going)	93.96	1007.707	0.720	0.917
Lack of energy	94.22	998.165	0.735	0.917
Dry skin	94.08	1013.112	0.553	0.920
Weight gain	95.58	1033.281	0.385	0.923
Increased facial hair	95.20	1020.736	0.648	0.918
Changes in appearance, texture or tone of my skin	95.34	1023.681	0.500	0.921
Frequent urination	95.17	1017.043	0.477	0.921
Involuntary urination when laughing or coughing	95.90	1035.548	0.381	0.923
Decrease in my sexual desire	94.37	997.512	0.586	0.919
Vaginal dryness	95.14	1016.225	0.467	0.921
Avoiding intimacy	94.97	1008.867	0.511	0.921

**Table 5:** Correlations between the MENQOL domains of menopausal women.

Domains	1	2	3	4
Vasomotor (1)	-			
Psychosocial (2)	0.451 **	-		
Physical (3)	0.367 **	0.525 **	-	
Sexual (4)	0.384 **	0.488 **	0.411 **	-
Global score	0.740 **	0.789 **	0.727 **	0.780 **

** *p* < 0.001.

**Table 6:** Sociodemographic variables and quality of life in menopausal women.

Variables		Domains				Global Score
		Vasomotor	Psychosocial	Physical	Sexual	
Age *		r = −0.018 *p* = 0.677	r = −0.175 *p* < 0.000	r = −0.138 *p* = 0.002	r = −0.095 *p* = 0.031	r = −0.133 *p* = 0.002
Marital status M (±SD)	Single	3.58 (2.31)	4.35 (1.85)	3.61 (1.72)	2.90 (2.12)	3.61 (1.56)
	Married/in a stable relationship	3.72 (2.04)	4.75 (1.66)	4.02 (1.66)	4.66 (2.16)	4.29 (1.42)
Statistical analysis		Z = −1.117 *p* = 0.264	Z = −1.847 *p* = 0.065	Z = −2.052 *p* = 0.040	Z = −6.869 *p* < 0.001	Z = −4.010 *p* < 0.001
Education M (±SD)	Primary [1]	4.60 (2.24)	5.04 (1.70)	4.58 (1.52)	4.90 (2.27)	4.78 (1.67)
	Secondary [2]	4.00 (1.89)	4.63 (1.64)	4.09 (1.54)	4.32 (2.50)	4.26 (1.420
	University degree [3]	3.44 (2.12)	4.65 (1.74)	3.81 (1.73)	4.27 (2.14)	4.04 (1.44)
Statistical analysis		H = 17.544 *p* < 0.001	H = 1.910 *p* = 0.385	H = 10.081 *p* = 0.006	H = 2.960 *p* = 0.228	H = 7.752 *p* = 0.021
Post hoc		1–3; 2–3	-	1–3	-	1–3
Professional status	Working	3.67 (2.14)	4.68 (1.68)	3.85 (1.66)	4.43 (2.24)	4.16 (1.45)
	Not working	3.76 (1.92)	4.64 (1.78)	4.29 (1.70)	4.01 (2.30)	4.18 (1.54)
Statistical analysis		Z = −0.777 *p* = 0.437	Z = −0.199 *p* = 0.842	Z = −2.501 *p* = 0.012	Z = −1.731 *p* = 0.083	Z = −0.197 *p* = 0.843
Material status **		rho = 0.120	rho = 0.036	rho = −0.017	rho = −0.063	rho = 0.024
		*p* = 0.006	*p* = 0.416	*p* = 0.706	*p* = 0.156	*p* = 0.580
BMI M (±SD)	Underweight [1]	3.00 (0.67)	6.98 (0.50)	5.42 (0.50)	3.25 (3.17)	4.66 (1.21)
	Normal weight [2]	3.27 (1.95)	4.68 (1.82)	3.46 (1.46)	4.53 (2.20)	3.98 (1.35)
	Overweight [3]	3.84 (2.05)	4.58 (1.47)	4.28 (1.64)	4.38 (2.28)	4.27 (1.44)
	Obese [4]	4.14 (2.29)	4.73 (1.88)	4.13 (1.91)	3.90 (2.26)	4.22 (1.69)
Statistical analysis		H = 13.644 *p* = 0.003	H = 8.259 *p* = 0.041	H = 28.271 *p* < 0.001	H = 5.997 *p* = 0.112	H = 3.634 *p* = 0.304
Post hoc		2−3; 2−4	1−3	2−3; 2−4	-	-
Impact on professional work M (±SD)	Yes	4.57 (2.13)	5.50 (1.42)	4.31 (1.63)	4.99 (2.29)	4.84 (1.27)
	No	3.05 (1.76)	3.95 (1.58)	3.63 (1.54)	4.08 (2.08)	3.68 (1.29)
Statistical analysis		Z = −7.084 *p* < 0.001	Z = −9.069 *p* < 0.001	Z = −4.136 *p* < 0.001	Z = −4.097 *p* < 0.001	Z = −8.190 *p* < 0.001
Impact on physical activity M (±SD)	Yes	4.52 (2.16)	5.38 (1.49)	4.39 (1.77)	5.09 (2.27)	4.84 (1.41)
	No	3.07 (1.69)	4.08 (1.62)	3.58 (1.44)	3.72 (2.08)	3.61 (1.20)
Statistical analysis		Z = −7.264 *p* < 0.001	Z = −8.006 *p* < 0.001	Z = −4.952 *p* < 0.001	Z = −6.324 *p* < 0.001	Z = −8.865 *p* < 0.001
Impact on social life M (±SD)	Yes	4.81 (2.10)	5.89 (1.39)	4.60 (1.75)	5.94 (1.83)	5.31 (1.19)
	No	3.37 (1.89)	4.24 (1.58)	3.71 (1.56)	3.77 (2.15)	3.77 (1.28)
Statistical analysis		Z = −6.999 *p* < 0.001	Z = −10.021 *p* < 0.001	Z = −5.128 *p* < 0.001	Z = −9.519 *p* < 0.001	Z = −10.989 *p* < 0.001

Note: * for age the Pearson’s r correlation coefficient was used; ** for marital status the rho Spearman rank correlation coefficient was used; r—Pearson correlation coefficient; Z—Mann–Whitney test; H—Kruskal–Wallis test; Post hoc—each row tests the null hypothesis that the Sample 1 and Sample 2 distributions are the same. The asymptotic significance (2-sided tests) are displayed. The significance values have been adjusted by the Bonferroni correction for multiple tests.

## Data Availability

The data presented in this study are available on request from the corresponding author.
